# Exposure to Non-Steroidal Anti-Inflammatory Drugs during Pregnancy and the Risk of Selected Birth Defects: A Prospective Cohort Study

**DOI:** 10.1371/journal.pone.0022174

**Published:** 2011-07-18

**Authors:** Marleen M. H. J. van Gelder, Nel Roeleveld, Hedvig Nordeng

**Affiliations:** 1 Department of Epidemiology, Biostatistics and HTA, Radboud University Nijmegen Medical Centre, Nijmegen, The Netherlands; 2 School of Pharmacy, University of Oslo, Oslo, Norway; 3 Division of Mental Health, Norwegian Institute of Public Health, Oslo, Norway; University of Giessen Lung Center, Germany

## Abstract

**Background:**

Since use of non-steroidal anti-inflammatory drugs (NSAIDs) during pregnancy is common, small increases in the risk of birth defects may have significant implications for public health. Results of human studies on the teratogenic risks of NSAIDs are inconsistent. Therefore, we evaluated the risk of selected birth defects after prenatal exposure to prescribed and over-the-counter NSAIDs.

**Methods and Findings:**

We used data on 69,929 women enrolled in the Norwegian Mother and Child Cohort Study between 1999 and 2006. Data on NSAID exposure were available from a self-administered questionnaire completed around gestational week 17. Information on pregnancy outcome was obtained from the Medical Birth Registry of Norway. Only birth defects suspected to be associated with NSAID exposure based upon proposed teratogenic mechanisms and previous studies were included in the multivariable logistic regression analyses. A total of 3,023 women used NSAIDs in gestational weeks 0–12 and 64,074 women did not report NSAID use in early pregnancy. No associations were observed between overall exposure to NSAIDs during pregnancy and the selected birth defects separately or as a group (adjusted odds ratio 0.7, 95% confidence interval 0.4–1.1). Associations between maternal use of specific types of NSAIDs and the selected birth defects were not found either, although an increased risk was seen for septal defects and exposure to multiple NSAIDs based on small numbers (2 exposed cases; crude odds ratio 3.9, 95% confidence interval 0.9–15.7).

**Conclusions:**

Exposure to NSAIDs during the first 12 weeks of gestation does not seem to be associated with an increased risk of the selected birth defects. However, due to the small numbers of NSAID-exposed infants for the individual birth defect categories, increases in the risks of specific birth defects could not be excluded.

## Introduction

Non-steroidal anti-inflammatory drugs (NSAIDs) are frequently used for their analgesic, antipyretic, and anti-inflammatory effects. They are among the most common drugs prescribed in the first trimester of pregnancy [1], and over-the-counter use of NSAIDs is also very widespread during pregnancy with prevalence estimates up to 19% [2], [3]. NSAIDs act as an inhibitor of cyclooxygenases (COXs), which catalyze the conversion of arachidonic acid to prostaglandins. Two isoforms of this enzyme have been identified: COX-1 and COX-2. The anti-inflammatory effects of NSAIDs are the result of COX-2 inhibition, while the adverse effects of non-selective NSAIDs are mainly due to the inhibition of COX-1 [4]. Results of animal studies suggest that COX-1 inhibition also may lead to cardiac, midline, and diaphragm defects [5], [6].

Since NSAID use during pregnancy is common, even small increases in the risk of birth defects may have significant implications for public health. Results of human studies on the teratogenic risks of first trimester NSAID use are inconsistent. Recent epidemiologic investigations showed an increased risk of congenital heart defects, especially cardiac septal defects, and orofacial clefts [7]–[9], while others did not find such effects [3], [10]. The aim of this study was to evaluate associations between maternal NSAID use during the first 12 weeks of gestation and the occurrence of selected birth defects using data from the Norwegian Mother and Child Cohort Study (MoBa), which includes information on both prescribed and over-the-counter NSAID use.

## Methods

### Ethics Statement

The study was approved by the Regional Committee for Medical Research Ethics and the Norwegian Data Inspectorate. All participants gave their written informed consent.

### Study population and data collection

MoBa is a prospective cohort study conducted by the Norwegian Institute of Public Health, which enrolled women in early pregnancy between 1999 and 2007. The objective of this study is to estimate the effects of a wide range of exposures during pregnancy on pregnancy outcome and maternal and child health [11]. During the enrollment period, participating hospitals and maternity units weekly provided lists of names and addresses of pregnant women living in Norway who requested routine ultrasound examination. The Norwegian Institute of Public Health subsequently sent these women a postal invitation to participate in MoBa, which included an information brochure, an informed consent form, and the first questionnaire, together with appointments for routine ultrasound scanning in gestational weeks 13–17. In this questionnaire, questions were asked about sociodemographic characteristics, maternal health, medication use, lifestyle factors, and occupational exposures during the 6 months prior to pregnancy and during the current pregnancy. The overall participation rate was 43.5% for pregnancies invited in MoBa [12]. To obtain information on pregnancy outcome, data from MoBa were linked to records in the Medical Birth Registry of Norway (MBRN) using the women's personal identification number. MBRN data are obtained by mandatory, standardized forms filled out by midwives, obstetricians, and/or pediatricians, and include detailed medical information regarding the health of both mother and newborn originating from medical records. All births that take place in Norway after gestational week 16 (after week 12 from 2002 onwards), including fetal deaths and elective terminations of pregnancy, are recorded in the MBRN [13]. For the current study, data were available for women enrolled in the period 1999–2006.

### Exposure and outcome definitions

Information on the type and timing of both prescribed and over-the-counter NSAID use was available from the questionnaire. If a woman reported use of an NSAID in the six months before or during pregnancy, she could specify five exposure windows: before pregnancy, gestational weeks 0–4, 5–8, 9–12, and 13+ (until completion of the first MoBa questionnaire). We defined NSAID exposure as use of any NSAID (Anatomical Therapeutic Classification code M01A or N02BA [14]) during gestational weeks 0–12. Women were considered non-exposed if they did not report use of any NSAIDs during pregnancy in the first MoBa questionnaire. In addition, the women were asked to report the number of days NSAIDs were taken. However, this question was completed by a minority of women and during the exploratory data analyses the data appeared to be highly unreliable. Therefore, they were not included in this study.

Only birth defects diagnosed by pediatricians and/or geneticists in the first week after birth or while the infants were in the hospital during their first year of life are included in the MBRN records. Birth defects are coded according to the *International Classification of Diseases*, *10^th^ Revision* (ICD-10) [15]. For this study, the outcome of interest was the presence of major birth defects that may result from NSAID exposure during pregnancy based upon proposed teratogenic mechanisms and previous epidemiologic studies [16]. These selected birth defects (ICD-10 code) included neural tube defects (Q00, Q01, and Q05), congenital heart defects, subdivided into conotruncal heart defects (Q20.0, Q20.1, Q20.3, Q21.3, Q21.4, and Q25.5–Q25.7 with Q21.0) and septal defects (Q21.0–Q21.2 and Q21.4), orofacial clefts (Q35–Q37), esophageal defects (Q39), anorectal malformations (Q42), diaphragmatic hernia (Q79.0), abdominal wall defects (Q79.2 and Q79.3), and amniotic bands (Q79.80). For classification of cases into isolated (no other major unrelated birth defect) and multiple birth defects (more than one unrelated major birth defect), the guidelines reported by Rasmussen et al were followed [17]. Infants without any major birth defect were considered unaffected.

### Statistical analysis

Our study population consisted of all women who completed the first MoBa questionnaire between 1999 and 2006 for whom data on pregnancy outcome from the MBRN were available (*n* = 69,929). Mothers with pre-existing diabetes were excluded from the analyses because of the known association between this condition and birth defects [18], [19]. Case infants with chromosomal abnormalities and mothers with multiple gestations or missing data on the timing of NSAID use (before or during pregnancy) were excluded as well.

Crude results were calculated as odds ratios (ORs) with 95% confidence intervals (CIs). We performed multivariable logistic regression analyses using a complete case analysis approach to estimate the risk of selected birth defects associated with NSAID exposure during the first 12 weeks of gestation, adjusted for maternal age at delivery (in years), maternal education (12 years or less vs. more than 12 years), parity (no previous live births vs. one or more previous live births), presence or absence of a history of miscarriages, stillbirths, or induced abortions, prepregnancy body-mass index (weight in kilograms divided by the square of height in meters; less than 25 vs. 25 or more), any maternal folic acid use from 4 weeks before pregnancy through week 8 of gestation, and fever and any maternal smoking during gestational weeks 0–12. Adjusted ORs were only calculated if at least three exposed cases were available. We used the same potential confounder set in all models, except when small numbers or a relatively high proportion of missing values (≥10% in either the group of affected or unaffected infants) prevented us from including one or more co-variables. In secondary analyses, we performed crude and adjusted analyses to assess the associations between the different types of NSAIDs (non-selective NSAIDs, acetic acid derivatives, and propionic acid derivatives) and four specific NSAIDs (diclofenac, ibuprofen, naproxen, and aspirin) and the occurrence of the selected birth defects. We also evaluated the effects of exposure to multiple NSAIDs during gestational weeks 0–12 on the risk of the selected birth defects. Additional analyses were performed on time window-specific exposure to NSAIDs. In sensitivity analyses, we assessed whether restricting the analyses to women without pre-existing diseases (asthma, hypertension, or epilepsy) or to infants with isolated defects only changed the effect estimates. Furthermore, we determined whether clustering due to enrollment in MoBa of multiple pregnancies by one woman influenced the results by including primiparae only, as we did not have information on the number of times a particular woman participated in MoBa. Finally, we estimated the potential effect of bias resulting from the relatively low response rate on the results. All statistical analyses were performed using SPSS version 17.0 for Windows (SPSS Inc., Chicago, IL.).

## Results

Of the 69,929 pregnant women with complete information, 2,038 had one or several of the following exclusion criteria: pre-existing diabetes (*n* = 390), diagnosis of a chromosomal abnormality (*n* = 121), multiple gestation (*n* = 1,290), and missing information on the timing of NSAID use (*n* = 256). Consequently, the final study population consisted of 67,891 women. A total of 6,972 (10.3%) women reported use of an NSAID in the 6 months before pregnancy and 3,023 (4.5%) women reported NSAID use during the first 12 weeks of gestation. Women who reported NSAID use in gestational weeks 13+ only (*n* = 569) and women who did not report the exact timing of NSAID use during pregnancy (*n* = 225) were omitted from further analyses. Ibuprofen was most commonly used (3.4%), followed by aspirin (0.5%), diclofenac (0.3%), and naproxen (0.2%). Most NSAID-using women used one NSAID (97.2%). Women who used NSAIDs during gestational weeks 0–12 were less likely to have more than 12 years of education, to be married or cohabiting, and to have had a previous live birth compared to non-using women (Table 1). NSAID-users were more likely than women who did not use NSAIDs during pregnancy to have had a previous miscarriage, stillbirth, or induced abortion, and to be overweight or obese. However, the absolute differences between the two groups were rather small.

**Table 1:** Characteristics of women who used and did not use non-steroidal anti-inflammatory drugs (NSAIDs) in gestational weeks 0–12.^a^

Characteristic	Subcategory	NSAID used (*n* = 3,023)	No NSAID used (*n* = 64,074)
Age at delivery	<20 y	42 (1.4)	689 (1.1)
	20–29 y	1,320 (43.7)	28,406 (44.3)
	30–39 y	1,607 (53.2)	33,774 (52.7)
	≥40 y	54 (1.8)	1,205 (1.9)
Education	<10 y	96 (3.2)	1,989 (3.1)
	10–12 y	1,134 (37.5)	21,575 (33.7)
	>12 y	1,639 (54.2)	37,109 (57.9)
	Other	62 (2.1)	1,091 (1.7)
	Missing	92 (3.0)	2,310 (3.6)
Married/cohabiting	Married/cohabiting	2,862 (94.7)	61,541 (96.0)
	Other	143 (4.7)	2,232 (3.5)
	Missing	18 (0.6)	301 (0.5)
Parity	0 previous live births	1,393 (46.1)	27,732 (43.3)
	≥1 previous live births	1,629 (53.9)	36,337 (56.7)
	Missing	1 (0.0)	5 (0.0)
Previous miscarriages, stillbirth, or induced abortions	None	1,874 (62.0)	41,175 (64.3)
	Miscarriage or stillbirth	572 (18.9)	11,661 (18.2)
	Induced abortion	401 (13.3)	7,190 (11.2)
	Induced abortion and miscarriage or stillbirth	123 (4.1)	2,347 (3.7)
	Missing	53 (1.8)	1,701 (2.7)
Prepregnancy body-mass index^b^	Underweight	74 (2.4)	1,947 (3.0)
	Normal weight	1,795 (59.4)	40,534 (63.2)
	Overweight	684 (22.6)	13,782 (21.5)
	Obese	398 (13.2)	5,917 (9.2)
	Missing	72 (2.4)	1,894 (3.0)
Any folic acid use^c^	Yes	1,916 (63.4)	40,611 (63.4)
	No	1,107 (36.6)	23,463 (36.6)
Pregnancy outcome	Live birth, still alive	2,993 (99.0)	63,622 (99.3)
	Live birth, died during follow-up	10 (0.3)	158 (0.2)
	Stillbirth	19 (0.6)	271 (0.4)
	Induced abortion	1 (0.0)	23 (0.0)

a Data from the Norwegian Mother and Child Cohort Study, 1999–2006. All data are presented as a number (%). Percentages may not add up to 100% due to rounding.

b The body-mass index is the weight in kilograms divided by the square of the height in meters: underweight: <18.5 kg/m^2^, normal weight: 18.5–24.9 kg/m^2^; overweight: 25.0–29.9 kg/m^2^, obese: ≥30 kg/m^2^.

c Folic acid use is reported from the 4 weeks prior to pregnancy through week 8 of gestation.

The prevalence of all major birth defects was 2.7% in our cohort (80 affected NSAID-exposed and 1,730 non-exposed infants). The selected birth defects were diagnosed in 638 infants (1.0%). A total of 18 infants had a neural tube defect (including 2 with anencephaly, 2 with encephalocele, and 15 with spina bifida), 435 a congenital heart defect (including 38 with conotruncal defect, 289 with ventricular septal defect, 156 with atrial septal defect, and 7 with atrioventricular septal defect), 134 an orofacial cleft (including 44 with cleft palate and 90 with cleft lip with or without cleft palate), 20 an esophageal defect, 16 an anorectal malformation, 11 a diaphragmatic defect, and 21 an abdominal wall defect (including 6 with omphalocele and 15 with gastroschisis). No infants in our study cohort were diagnosed with amniotic bands. Of the infants with selected birth defects, 42 infants (6.6%) were classified as having multiple defects. Our study cohort included 65,287 infants without a major birth defect.

Table S1 shows the characteristics of the 23 infants exposed to NSAIDs in the first 12 weeks of gestation who had any of the selected birth defects. The maternal age at delivery ranged from 25 to 35 years. A total of 21 infants were live born at gestational ages ranging from 34 to 42 weeks, one woman had a miscarriage at 18 weeks of gestation, and one infant was stillborn at a gestational age of 39 weeks. All but five of these infants were exposed to other medications during pregnancy in addition to NSAIDs, of which one infant was exposed to a drug generally considered teratogenic (podophyllotoxin).

The crude and adjusted odds ratios for overall NSAID exposure and the selected birth defects are shown in Table 2. Any NSAID use during the first 12 weeks of gestation was not associated with all selected birth defects as a group (adjusted OR 0.7 (95% CI 0.4–1.1)) nor with any of the birth defect categories, including any congenital heart defects (adjusted OR 0.9 (95% CI 0.5–1.4)), septal defects (adjusted OR 0.8 (95% CI 0.5–1.4)), ventricular septal defects (adjusted OR 0.7 (95% CI 0.4–1.4)), and atrial septal defects (adjusted OR 1.1 (95% CI 0.5–2.3)). For the other groups of birth defects, there were too few exposed cases to reliably estimate adjusted odds ratios. A crude OR of 0.2 (95% CI 0.0–1.1) was seen for orofacial clefts based on one exposed case. Analyses for the three exposure time-windows assessed in the questionnaire separately did not alter these results (Table 3), although we saw a slightly increased risk for atrial septal defects after NSAID exposure in gestational weeks 5–8 (adjusted OR 1.6 (95% CI 0.7–3.9)).

**Table 2:** Associations between maternal use of non-steroidal anti-inflammatory drugs (NSAIDs) in gestational weeks 0–12 and selected birth defects.^a^

			NSAID used (*n* = 3,023)	No NSAID used (*n* = 64,074)	Odds ratio (95% CI)	
Birth defect	Subcategory 1	Subcategory 2	*n* (%)	*n* (%)	Crude	Adjusted^b^
No major birth defects			2,943 (97.4)	62,344 (97.3)	Reference	Reference
All selected birth defects			23 (0.8)	615 (1.0)	0.8 (0.5–1.2)	0.7 (0.4–1.1)
Neural tube defects			1 (0.0)	17 (0.0)	1.2 (0.2–9.4)	–
Congenital heart defects			20 (0.7)	415 (0.6)	1.0 (0.7–1.6)	0.9 (0.5–1.4)
	Conotruncal heart defects		2 (0.1)	36 (0.1)	1.2 (0.3–4.9)	–
	Septal defects		18 (0.6)	394 (0.6)	1.0 (0.6–1.6)	0.8 (0.5–1.4)
		Ventricular septal defect	11 (0.4)	278 (0.4)	0.8 (0.5–1.5)	0.7 (0.4–1.4)
		Atrial septal defect	8 (0.3)	148 (0.2)	1.1 (0.6–2.3)	1.1 (0.5–2.3)^c^
Orofacial clefts			1 (0.0)	133 (0.2)	0.2 (0.0–1.1)	–
Esophageal defects			0 (0.0)	20 (0.0)	–	–
Anorectal malformations			1 (0.0)	15 (0.0)	1.4 (0.2–10.7)	–
Diaphragmatic hernia			0 (0.0)	11 (0.0)	–	–
Abdominal wall defects			0 (0.0)	21 (0.0)	–	–
Amniotic bands			0 (0.0)	0 (0.0)	–	–

a Data from the Norwegian Mother and Child Cohort Study, 1999–2006. Infants with multiple selected birth defects were included in all relevant outcome categories. Adjusted analyses were performed if at least three exposed cases were available.

b Adjusted for maternal age at delivery, education, parity, history of miscarriages, stillbirths, or induced abortions, prepregnancy body-mass index, folic acid use, fever, and smoking.

c Adjusted for maternal age at delivery, parity, prepregnancy body-mass index, folic acid use, and smoking.

**Table 3:** Associations between maternal use of non-steroidal anti-inflammatory drugs (NSAIDs) during the three exposure windows and selected birth defects.^a^

			No NSAID used (*n* = 64,074)	NSAID used in gestational weeks 0–4 (*n* = 1,607)		NSAID used in gestational weeks 5–8 (*n* = 1,329)		NSAID used in gestational weeks 9–12 (*n* = 1,422)	
Birth defect	Subcategory 1	Subcategory 2	*n*	*n*	Adjusted odds ratio (95% CI)^b^	*n*	Adjusted odds ratio (95% CI)^c^	*n*	Adjusted odds ratio (95% CI)^d^
No major birth defects			62,344	1,561	Reference	1,290	Reference	1,383	Reference
All selected birth defects			615	10	0.7 (0.4–1.3)	12	0.8 (0.4–1.5)	10	0.7 (0.4–1.3)
Neural tube defects			17	1	–	0	–	1	–
Congenital heart defects			415	9	0.9 (0.5–1.8)^e^	11	1.0 (0.5–2.0)	8	0.9 (0.4–1.8)^f^
	Conotruncal heart defects		36	1	–	1	–	2	–
	Septal defects		394	8	0.9 (0.4–1.7)^e^	10	0.9 (0.4–1.9)	6	0.7 (0.3–1.6)^f^
		Ventricular septal defect	278	6	0.9 (0.4–2.0)^e^	6	1.1 (0.5–2.5)^g^	2	–
		Atrial septal defect	148	3	0.8 (0.3–2.7)^h^	5	1.6 (0.7–3.9)^i^	4	1.2 (0.4–3.3)^f^
Orofacial clefts			133	0	–	0	–	1	–
Anorectal malformations			15	0	–	1	–	0	–

a Data from the Norwegian Mother and Child Cohort Study, 1999–2006. Infants with multiple selected birth defects were included in all relevant outcome categories. Adjusted analyses were performed if at least three exposed cases were available.

b Adjusted for maternal age at delivery, education, parity, history of miscarriages, stillbirths, or induced abortions, prepregnancy body-mass index, folic acid use, and fever.

c Adjusted for maternal age at delivery, education, parity, history of miscarriages, stillbirths, or induced abortions, prepregnancy body-mass index, folic acid use, fever, and smoking.

d Adjusted for maternal age at delivery, parity, prepregnancy body-mass index, folic acid use, and fever.

e Adjusted for maternal age at delivery, education, parity, history of miscarriages, stillbirths, or induced abortions, prepregnancy body-mass index, and folic acid use.

f Adjusted for maternal age at delivery, parity, prepregnancy body-mass index, and folic acid use.

g Adjusted for maternal age at delivery, education, parity, history of miscarriages, stillbirths, or induced abortions, folic acid use, fever, and smoking.

h Adjusted for maternal age at delivery, education, parity, prepregnancy body-mass index, and folic acid use.

i Adjusted for maternal age at delivery, parity, prepregnancy body-mass index, folic acid use, and smoking.

We conducted several secondary analyses to evaluate the effects of exposure to different types of NSAIDs on the occurrence of the selected birth defects (Table 4). Restricting the exposed group to infants exposed to non-selective NSAIDs (excluding infants exposed to coxibs only) did not change the results of the primary analyses. No associations were observed between the selected birth defects as a group and exposure to acetic acid derivatives (1 exposed case), propionic acid derivatives (adjusted OR 0.7 (95% CI 0.4–1.2)), aspirin (adjusted OR 1.1 (95% CI 0.4–3.5)), or multiple NSAIDs (crude OR 2.5 (95% CI 0.6–10.1)). However, we found an association between exposure to multiple NSAIDs during gestational weeks 0–12 and congenital heart defects (crude OR 3.7 (95% CI 0.9–14.9)), in particular septal defects (crude OR 3.9 (95% CI 0.9–15.7)), but this observation was based on only 2 exposed case infants. We did not find associations between any of the other NSAID subgroups and congenital heart defects or septal defects.

**Table 4:** Secondary analyses of the risk of selected birth defects among infants with exposure to non-steroidal anti-inflammatory drugs (NSAIDs) in gestational weeks 0–12.^a^

			Any selected birth defect		Congenital heart defect		Septal defects	
NSAID exposure	Subcategory	Total *n*	*n* (%)	Adjusted odds ratio (95% CI)^b^	*n* (%)	Adjusted odds ratio (95% CI)^b^	*n* (%)	Adjusted odds ratio (95% CI)^b^
None		64,074	615 (1.0)	Reference	415 (0.6)	Reference	394 (0.6)	Reference
Non-selective NSAIDs		2,964	23 (0.8)	0.7 (0.4–1.1)	20 (0.7)	0.9 (0.5–1.5)	18 (0.6)	0.8 (0.5–1.4)
Acetic acid derivatives		189	1 (0.5)	–	1 (0.5)	–	1 (0.5)	–
	Diclofenac	169	1 (0.6)	–	1 (0.6)	–	1 (0.6)	–
Propionic acid derivatives		2,425	19 (0.8)	0.7 (0.4–1.2)^c^	16 (0.7)	0.8 (0.5–1.4)	14 (0.6)	0.7 (0.4–1.3)
	Ibuprofen	2,276	19 (0.8)	0.8 (0.5–1.3)^c^	16 (0.7)	0.9 (0.5–1.5)	14 (0.6)	0.7 (0.4–1.4)
	Naproxen	166	0 (0.0)	–	0 (0.0)	–	0 (0.0)	–
Aspirin		307	3 (1.0)	1.1 (0.4–3.5)^d^	3 (1.0)	1.6 (0.5–5.2)^d^	3 (1.0)	1.7 (0.6–5.4)^d^
Multiple NSAIDs		86	2 (2.3)	–	2 (2.3)	–	2 (2.3)	–

a Data from the Norwegian Mother and Child Cohort Study, 1999–2006. Infants with multiple selected birth defects were included in all relevant outcome categories. Adjusted analyses were performed if at least three exposed cases were available.

b Adjusted for maternal age at delivery, education, parity, history of miscarriages, stillbirths, or induced abortions, prepregnancy body-mass index, folic acid use, fever, and smoking.

c Adjusted for maternal age at delivery, education, parity, history of miscarriages, stillbirths, or induced abortions, folic acid use, fever, and smoking.

d Adjusted for maternal age at delivery, education, parity, prepregnancy body-mass index, and folic acid use.

The detailed results of the sensitivity analyses are shown in Appendix S1. Analyses restricted to women without pre-existing diseases did not alter the results of the primary analyses, nor did restricting the analyses to affected infants with isolated birth defects only. Restricting the primary analyses to primiparae to estimate the effect of clustering due to enrollment in MoBa of multiple pregnancies by one woman did not change the effect estimates substantially, but it did decrease precision. The sensitivity analysis to estimate the potential bias resulting from the relatively low response rate indicated that selective participation of mothers of either exposed, non-exposed, affected, or unaffected infants did not change the effect estimates of the primary analysis. Only in the unlikely event that one of the four exposure-outcome groups was far more likely to participate in MoBa than the other three groups, the NSAID-birth defect associations observed could have been biased due to selection.

## Discussion

In this large prospective cohort study, we did not find associations between exposure to any NSAID in the first 12 weeks of gestation and the occurrence of birth defects such as congenital heart defects and orofacial clefts, which were selected based upon results of previous animal and epidemiologic studies and the proposed teratogenic mechanism of NSAIDs. However, we did observe a non-statistically significantly increased risk of septal defects after exposure to multiple NSAIDs in the first 12 weeks of gestation. Furthermore, it should be kept in mind that NSAIDs have been associated with spontaneous abortions [10], [20] and that they are contraindicated during the third trimester of pregnancy due to an increased risk of premature closure of the ductus arteriosus [21], [22].

Although NSAID use during pregnancy is prevalent, epidemiologic studies on the teratogenic risks are relatively sparse. Recent case-control studies, which may be prone to recall bias, showed possible associations between NSAID exposure and ventricular septal defects [23], amniotic bands [24], and gastroschisis [25]. Two cohort studies using data from registries, of which one used reports from the first prenatal care visit and the other lacked information on compliance and over-the-counter use of NSAIDs, generally found no increased risks of birth defects after NSAID exposure [7], [10], but associations with congenital heart defects and orofacial clefts were reported in the former [7]. A third pregnancy register showed increased risks of any birth defect and of septal defects in particular among infants of women who filled a prescription for NSAIDs in the first trimester [9]. In a recent study which used follow-up data of women who contacted Teratology Information Services, an increased risk of major birth defects after exposure to diclofenac in gestational weeks 5–14 could not be excluded (crude OR 2.5 (95% CI 0.9–6.6)) [26]. To our knowledge, the current study, using data collected in the Norwegian Mother and Child Cohort Study, is the first prospective cohort study to evaluate the teratogenic risk of both prescribed and over-the-counter NSAIDs, thereby avoiding differential misclassification by the outcome of interest often found in retrospective studies.

In secondary analyses, we observed a possible association between exposure to multiple NSAIDs and septal defects with two (2.3%) of the exposed infants being diagnosed with either a ventricular or an atrial septal defect. This finding was based on very small numbers, but there are several reasons why this finding might indicate a truly increased risk. First, all outcomes of interest, including septal defects, were selected based on biologic plausibility. Secondly, mothers of exposed cases used a combination of either ibuprofen and ketoprofen or ibuprofen and diclofenac, which all inhibit COX-1 by more than 60% when COX-2 is inhibited by 80% [27]. Animal studies indicate that especially NSAIDs with a high COX-1/COX-2 ratio may cause birth defects [5], in particular since COX-1 is expressed in rat embryos during cardiovascular development [28]. Finally, both animal and human studies have indicated an increased risk of septal defects after prenatal NSAID exposure [9], [20], [29]. However, as cardiac septation takes place between weeks 4 and 7 of development [30], exposure did not occur during the etiologically relevant period for one of the exposed cases (Table S1). Therefore, these results should be interpreted with caution and confirmation by other studies is warranted.

The absence of associations between maternal use of NSAIDs during pregnancy and the occurrence of the selected birth defects may partly be due to the relatively low prevalence of exposure in this cohort (4.5%). Studies conducted in the U.S. found much higher prevalence estimates of NSAID use during the first trimester up to approximately 19% [2], [3]. Comparable European data are lacking, but recent reports indicate that NSAID use during pregnancy may be less prevalent in European countries compared to the U.S. [31], [32], especially since NSAIDs are contraindicated in the first and third trimester of pregnancy. In a study using data from the Norwegian Prescription Database, 2.0% of women filled at least one NSAID prescription in the first trimester of pregnancy [33]. Therefore, we believe that the prevalence of NSAID use during the first 12 weeks of gestation found in our study population is accurate. However, the small number of NSAID-exposed infants for the individual birth defect categories remains a limitation, which made lumping of birth defects necessary for power purposes, which, in turn, may have masked true associations between prenatal NSAID exposure and specific birth defects.

The main strength of this study is its longitudinal design which features prospective ascertainment of NSAID use and other covariate information obtained at a median of 17 weeks of gestation. However, as MoBa has a relatively low participation rate (43.5%), selection bias may have occurred. A recent non-response study showed that the prevalence estimates of several exposures and birth outcomes are biased in MoBa, but that estimates of exposure-outcome associations are not biased due to self-selection [12]. Similar results were obtained from a comparable cohort study conducted in Denmark [34]. In addition, the sensitivity analyses showed that selective participation on either exposure or disease status of the infant did not influence our effect estimates. The only scenario in the sensitivity analyses that affected our NSAID-birth defect risk estimates was when one of the four exposure-outcome groups was more likely to participate than the other three groups, but this is highly unlikely in a prospective cohort study. Therefore, we feel that our results on the associations between prenatal NSAID exposure and selected birth defects are not biased by the low participation rate.

Non-differential misclassification of the exposure status may have occurred since data on NSAID use were collected using self-administered questionnaires. In addition, there might be a chance that the lack of an increased risk of the selected birth defects in NSAID-exposed infants is due to the inability to separate occasional users from the more frequent or continuous users. Confounding by indication cannot be excluded completely either, although restricting our analyses to women without pre-existing diseases did not change the results of the primary analyses, in which fever in the first 12 weeks of gestation was also included as a potential confounder.

Several validation studies have been conducted regarding the accuracy of the MBRN, which showed that the ascertainment of congenital malformations varies according to the type of defect and its severity. For the years 2001–2005, 82% of clinically verified cases of Down syndrome were recorded in the registry [35]. Overall, 71% of cases with severe isolated cleft palate were reported, whereas as little as 11% of cases with mild cleft palate were recorded [36]. The registration of cardiovascular malformations in the MBRN has not been validated nor confirmed by a geneticist or dysmorphologist. Therefore, misclassification of the outcomes of interest may have occurred in the current study, but we do not expect the ascertainment rates to differ between infants who were exposed to NSAIDs in pregnancy and non-exposed infants. However, this may have decreased our study power and may have led to underestimation of our effect estimates, although the total prevalence of major birth defects in our cohort (2.7%) is comparable to the expected prevalence of 3% in most populations.

### Conclusions

The findings of this large prospective cohort study showed no associations between exposure to NSAIDs during the first part of pregnancy and the risk of selected birth defects, although an increased odds ratio was seen for septal defects after exposure the multiple NSAIDs. This observation, based on only two exposed cases, was not statistically significant and needs confirmation by other studies. However, due to the small numbers of NSAID-exposed infants for the individual birth defect categories, increases in the risks of specific birth defects could not be excluded.

## Supporting Information

Table S1 Characteristics of the 23 infants born with selected birth defects after prenatal exposure to non-steroidal anti-inflammatory drugs (NSAIDs).
(PDF)

Appendix S1 Results of the sensitivity analyses on the effect of prenatal exposure to non-steroidal anti-inflammatory drugs (NSAIDs) and selected birth defects.
(PDF)
