# Supplementary material for: Exposure to Non-Steroidal Anti-Inflammatory Drugs during Pregnancy and the Risk of Selected Birth Defects: A Prospective Cohort Study
Source: PLoS One. 2011 Jul 18;6(7):e22174. doi: 10.1371/journal.pone.0022174 (PMC3138772; doi:10.1371/journal.pone.0022174)
Supplement: Table S1 — Characteristics of the 23 infants born with selected birth defects after prenatal exposure to non-steroidal anti-inflammatory drugs (NSAIDs). (PDF) [file pone.0022174.s001.pdf]

Table S1. Characteristics of the 23 infants born with selected birth defects after prenatal exposure to non-steroidal anti-inflammatory drugs (NSAIDs).

Birth defect	Maternal age (y)	Gestational age (wk)	Pregnancy outcome	NSAID used	Timing of exposure (gest. wk)	Chronic/serious maternal condition	Other drugs used in first part of pregnancy
Anencephaly, spina bifida	32	18	Miscarriage	Ibuprofen	0–4, 9–12	None	Acetaminophen, ebastine, fluticasone, unspecified allergy/asthma medication
Atrial septal defect	30	39	Live birth	Aspirin	9–12	Endocarditis	Dermatological preparation for hemorrhoids
Atrial septal defect	28	37	Live birth	Ibuprofen	5–12	None	Acetaminophen
Atrial septal defect	33	41	Live birth	Ibuprofen	0–4	None	None
Atrial septal defect	31	37	Live birth	Ibuprofen, ketoprofen	5–12	Cardiopathy	Clotrimazole, xylometazoline
Atrial septal defect	35	40	Live birth	Ibuprofen	5–12	None	Acetaminophen
Atrial septal defect, patent ductus arteriosus	34	37	Live birth	Piroxicam	5–8	None	Acetaminophen, phenoxymethylpenicillin
Atrial septal defect, patent ductus arteriosus	27	40	Live birth	Ibuprofen	0–4	None	Cetirizine
Atrial and ventricular septal defects, patent ductus arteriosus	28	39	Live birth	Aspirin	0–8	None	Acetaminophen, alginic acid
Cleft lip	28	40	Live birth	Ibuprofen	9–12	None	None
Imperforate anus	30	42	Live birth	Ibuprofen	5–8	None	Econazole
Tetralogy of Fallot, other anomaly pulmonary artery	31	34	Live birth	Ibuprofen	9–12	None	Cyclizine, oxymetazoline
Transposition of the great vessels, coarctation of aorta	35	39	Stillbirth	Ibuprofen	0–12	None	None
Ventricular septal defect	28	41	Live birth	Ibuprofen	5–8	None	Benzoyl peroxide, budesonide, phenylpropanolamine, podophyllotoxin
Ventricular septal defect	35	40	Live birth	Ibuprofen	5–8	Epilepsy	Lamotrigine, sumatriptan
Ventricular septal defect	27	40	Live birth	Ibuprofen	0–12	None	Acetaminophen

Ventricular septal defect	29	38	Live birth	Aspirin	0–4	Asthma, ventricular tachycardia	Acetaminophen, flecainide, hydrocortisone, unspecified dermatologic preparation
Ventricular septal defect	31	40	Live birth	Ibuprofen	5–8	None	Ferrous sulfate
Ventricular septal defect	32	42	Live birth	Ibuprofen	9–12	None	Acetaminophen, clomitrazole, nitrofurantoin, pivmecillinam
Ventricular septal defect	32	40	Live birth	Diclofenac, ibuprofen	0–4	None	Bumetanide, clotrimazole, lactulose
Ventricular septal defect	25	38	Live birth	Ibuprofen	0–4	Epilepsy	None
Ventricular septal defect	33	41	Live birth	Ibuprofen	5–8	None	None
Ventricular septal defect, patent ductus arteriosus	28	40	Live birth	Ibuprofen	0–4	Asthma, chronic urinary tract infections	Acetaminophen, amitriptyline, clotrimazole, desonide, tramadol
