# Supplementary material for: Exposure to Non-Steroidal Anti-Inflammatory Drugs during Pregnancy and the Risk of Selected Birth Defects: A Prospective Cohort Study
Source: PLoS One. 2011 Jul 18;6(7):e22174. doi: 10.1371/journal.pone.0022174 (PMC3138772; doi:10.1371/journal.pone.0022174)
Supplement: Appendix S1 — Results of the sensitivity analyses on the effect of prenatal exposure to non-steroidal anti-inflammatory drugs (NSAIDs) and selected birth defects. (PDF) [file pone.0022174.s002.pdf]

Appendix S1. Results of the sensitivity analyses on the effect of prenatal exposure to non-steroidal anti-inflammatory drugs (NSAIDs) and selected birth defects.

A. Excluding women with pre-existing diseases

The main analyses were restricted to women without pre-existing diseases, excluding those women who reported having asthma (*n*=4,992), hypertension (*n*=673), or epilepsy (*n*=439) before the index pregnancy (total *n*=6,007). A total of 61,884 women without pre-existing diseases were included in sensitivity analysis A, of which the results are shown in Table A.

Table A. Associations between maternal use of NSAIDs and selected birth defects among women without pre-existing diseases.[a]

**Birth defect**	**NSAID used (*n*=2,650) *n* (%)**	**No NSAID used (*n*=58,437) *n* (%)**	**Odds ratio (95% CI) Crude**	**Adjusted[b]**
No major birth defects	2,650 (97.6)	56,872 (97.3)	Reference	Reference
All selected birth defects	18 (0.7)	549 (0.9)	0.7 (0.4–1.1)	0.6 (0.4–1.0)[c]
Neural tube defects	1 (0.0)	14 (0.0)	1.5 (0.2–11.7)	–
Congenital heart defects	16 (0.6)	372 (0.6)	0.9 (0.6–1.5)	0.7 (0.4–1.3)
Conotruncal heart defects	2 (0.1)	32 (0.1)	1.3 (0.3–5.6)	–
Septal defects	14 (0.5)	353 (0.6)	0.9 (0.5–1.5)	0.6 (0.3–1.2)
Ventricular septal defect	7 (0.3)	254 (0.4)	0.6 (0.3–1.3)	0.6 (0.3–1.2)[d]
Atrial septal defect	8 (0.3)	129 (0.2)	1.3 (0.7–2.7)	1.3 (0.6–2.6)[e]
Orofacial clefts	1 (0.0)	119 (0.2)	0.2 (0.0–1.3)	–
Esophageal defects	0 (0.0)	19 (0.0)	–	–
Anorectal malformations	0 (0.0)	15 (0.0)	–	–
Diaphragmatic hernia	0 (0.0)	6 (0.0)	–	–
Abdominal wall defects	0 (0.0)	19 (0.0)	–	–

[a]Data from the Norwegian Mother and Child Cohort Study, 1999–2006. Infants with multiple selected birth defects were included in all relevant outcome categories. Adjusted analyses were performed if at least three exposed cases were available.

[b]Adjusted for maternal age at delivery, education, parity, history of miscarriages, stillbirths or induced abortions, prepregnancy body-mass index, folic acid use, fever, and smoking.

[c]Adjusted for maternal age at delivery, education, parity, history of miscarriages, stillbirths or induced abortions, folic acid use, fever, and smoking.

[d]Adjusted for maternal age at delivery, education, parity, history of miscarriages, stillbirths or induced abortions, folic acid use, and fever.

[e]Adjusted for maternal age at delivery, parity, prepregnancy body-mass index, folic acid use, and smoking.

B. Restriction to infants with isolated defects only

The main analyses were restricted to case infants with isolated defects only, excluding infants who were diagnosed with multiple defects (*n*=42). A total of 67,055 women were included in sensitivity analysis B. The results are shown in Table B.

Table B. Associations between maternal use of NSAIDs and selected isolated birth defects.[a]

Birth defect	NSAID used (*n*=3,023) *n* (%)	No NSAID used (*n*=64,032) *n* (%)	Odds ratio (95% CI) Crude	Adjusted[b]
No major birth defects	2,943 (97.4)	62,344 (97.4)	Reference	Reference
All selected birth defects	23 (0.8)	573 (0.9)	0.9 (0.6–1.3)	0.7 (0.5–1.2)
Neural tube defects	1 (0.0)	15 (0.0)	1.4 (0.2–10.7)	–
Congenital heart defects	20 (0.7)	387 (0.6)	1.1 (0.7–1.7)	0.9 (0.6–1.5)
Conotruncal heart defects	2 (0.1)	30 (0.0)	1.4 (0.3–5.9)	–
Septal defects	18 (0.6)	370 (0.6)	1.0 (0.6–1.7)	0.9 (0.5–1.5)
Ventricular septal defect	11 (0.4)	266 (0.4)	0.9 (0.5–1.6)	0.8 (0.4–1.5)
Atrial septal defect	8 (0.3)	134 (0.2)	1.3 (0.6–2.6)	1.2 (0.6–2.5)[c]
Orofacial clefts	1 (0.0)	124 (0.2)	0.2 (0.0–1.2)	–
Esophageal defects	0 (0.0)	11 (0.0)	–	–
Anorectal malformations	1 (0.0)	10 (0.0)	2.1 (0.3–16.6)	–
Diaphragmatic hernia	0 (0.0)	9 (0.0)	–	–
Abdominal wall defects	0 (0.0)	19 (0.0)	–	–

[a]Data from the Norwegian Mother and Child Cohort Study, 1999–2006. Infants with multiple selected birth defects were included in all relevant outcome categories. Adjusted analyses were performed if at least three exposed cases were available.

[b]Adjusted for maternal age at delivery, education, parity, history of miscarriages, stillbirths, or induced abortions, prepregnancy body-mass index, folic acid use, fever, and smoking.

[c]Adjusted for maternal age at delivery, parity, prepregnancy body-mass index, folic acid use, and smoking.

C. Restriction to primiparae only

Among women enrolled between 1999–2005, 90.7% participated with one pregnancy, 9.0% with two pregnancies, 0.3% with three pregnancies, and one woman with four pregnancies.[11] However, as we did not have information on which women participated multiple times in MoBa, we included only primiparae in this sensitivity analysis to estimate the potential effect of clustering due to enrollment of multiple pregnancies by one woman. A total of 29,471 primiparae were included in sensitivity analysis C. The results are displayed in Table C.

Table C. Associations between maternal use of NSAIDs and selected birth defects among primiparae.[a]

**Birth defect**	**NSAID used (*n*=1,393)**	**No NSAID used (*n*=27,732)**	**Odds ratio (95% CI)**	
	***n* (%)**	***n* (%)**	**Crude**	**Adjusted[b]**
No major birth defects	1,355 (97.3)	26,884 (96.9)	Reference	Reference
All selected birth defects	9 (0.6)	291 (1.0)	0.6 (0.3–1.2)	0.6 (0.3–1.2)
Neural tube defects	0 (0.0)	9 (0.0)	–	–
Congenital heart defects	7 (0.5)	200 (0.7)	0.7 (0.3–1.5)	0.8 (0.4–1.6)
Conotruncal heart defects	0 (0.0)	20 (0.1)	–	–
Septal defects	7 (0.5)	188 (0.7)	0.7 (0.3–1.6)	0.8 (0.4–1.7)
Ventricular septal defect	5 (0.4)	134 (0.5)	0.7 (0.3–1.8)	0.8 (0.3–1.9)
Atrial septal defect	3 (0.2)	73 (0.3)	0.8 (0.3–2.6)	0.9 (0.3–2.8)[c]
Orofacial clefts	1 (0.1)	57 (0.2)	0.3 (0.0–2.5)	–
Esophageal defects	0 (0.0)	12 (0.0)	–	–
Anorectal malformations	1 (0.1)	8 (0.0)	2.5 (0.3–19.8)	–
Diaphragmatic hernia	0 (0.0)	4 (0.0)	–	–
Abdominal wall defects	0 (0.0)	14 (0.1)	–	–

[a]Data from the Norwegian Mother and Child Cohort Study, 1999–2006. Infants with multiple selected birth defects were included in all relevant outcome categories. Adjusted analyses were performed if at least three exposed cases were available.

[b]Adjusted for maternal age at delivery, education, prepregnancy body-mass index, and folic acid use.

[c]Adjusted for maternal age at delivery and education.

D. Potential effect of bias due to relatively low response rate

Participation rates of less than 100% may introduce selection bias if the expose-disease association is different for participants than for all subjects eligible for inclusion in the study. The participation rate for MoBa was 43.5%, so our target population included 154,246 subjects. Because we did not have prevalence data on NSAID use in the first 12 weeks of gestation and on the selected birth defects among infants eligible for inclusion in our study cohort, we assessed the potential effect of selection bias in a simulation study. The results of this analysis are shown in Table D.

Table D. Potential effect of selection bias on the crude effect estimates on the association between prenatal exposure to non-steroidal anti-inflammatory drugs (NSAIDs) and selected birth defects.

	Participation rate (%) among				Crude OR (95% CI) in target population (*n*=154,246)		
**Scenario**	**Exposed affected**	**Non-exposed affected**	**Exposed unaffected**	**Non-exposed unaffected**	**Any selected birth defect**	**Congenital heart defect**	**Atrial septal defect**
1.	43.5	43.5	43.5	43.5	0.8 (0.6–1.0)	1.0 (0.8–1.4)	1.1 (0.7–1.8)
2.	85.0	42.5	85.0	42.5	0.8 (0.5–1.2)	1.0 (0.7–1.6)	1.1 (0.6–2.1)
3.	22.7	45.5	22.7	45.5	0.8 (0.6–1.0)	1.0 (0.8–1.3)	1.1 (0.8–1.6)
4.	86.6	86.6	43.3	43.3	0.8 (0.5–1.2)	1.0 (0.7–1.5)	1.1 (0.6–2.2)
5.	22.0	22.0	43.9	43.9	0.8 (0.7–1.0)	1.0 (0.8–1.3)	1.1 (0.8–1.6)
6.	87.0	43.5	43.5	43.5	0.4 (0.3–0.6)	0.5 (0.3–0.8)	0.6 (0.3–1.1)
7.	43.3	86.6	43.3	43.3	1.6 (1.2–2.1)	2.0 (1.5–2.8)	2.2 (1.4–3.6)
8.	42.5	42.5	85.0	42.5	1.6 (1.2–2.1)	2.0 (1.5–2.7)	2.3 (1.5–3.7)
9.	22.8	22.8	22.8	45.9	0.4 (0.3–0.5)	0.5 (0.4–0.6)	0.5 (0.4–0.7)

Scenarios:
1. No selection bias.
2. NSAID-using women were twice as likely to participate compared to non-using women.
3. Non-using women were twice as likely to participate compared to NSAID-using women.
4. Mothers of affected infants were twice as likely to participate compare to mothers of unaffected infants.
5. Mothers of unaffected infants were twice as likely to participate compared to mothers of affected infants.
6. Mothers of exposed affected infants were twice as likely to participate compared to the other groups.
7. Mothers of non-exposed affected infants were twice as likely to participate compared to the other groups.
8. Mothers of exposed unaffected infants were twice as likely to participate compared to the other groups.
9. Mothers of non-exposed unaffected infants were twice as likely to participate compared to the other groups.
